# An audit of the iron status of patients at Chris Hani Baragwanath Academic Hospital, in Johannesburg, South Africa

**DOI:** 10.4102/ajlm.v13i1.2509

**Published:** 2024-10-18

**Authors:** Jurette S. Grove, Siyabonga Khoza, Dineo V. Mabuza, Shaida B. Khan

**Affiliations:** 1 Department of Chemical Pathology, Faculty of Health Sciences, University of the Witwatersrand, Johannesburg, South Africa; 2 National Health Laboratory Service, Johannesburg, South Africa; 3 Department of Chemical Pathology, Faculty of Pathology, PathCare Laboratories, Johannesburg, South Africa

**Keywords:** Iron, iron status, ferritin, ferritin diagnostic threshold, iron deficiency, iron deficiency anaemia, anaemia of chronic disease

## Abstract

**Background:**

Iron deficiency is a common disorder, especially in developing countries. Accurately assessing iron status remains challenging, particularly for patients with chronic diseases such as HIV and chronic kidney disease, prevalent in South Africa.

**Objective:**

This study aimed to determine how ferritin cut-offs affect iron status classification in adult patients treated at a tertiary hospital in South Africa. Additionally, it assessed the frequency of these conditions and the impact of age and gender on iron status.

**Methods:**

This retrospective study analysed iron profiles in adult patients from 01 October 2020 to 31 March 2021. Iron status was categorised into five groups: iron deficiency anaemia (IDA), anaemia of chronic disease, IDA with anaemia of chronic disease, iron deficiency without anaemia, and iron replete based on haemoglobin, transferrin saturation, and ferritin levels. The impact of using two different ferritin cut-off values (15 µg/L and 30 µg/L) was investigated.

**Results:**

The study included 3221 complete iron profiles. There was a predominance of female patients (2.2:1 ratio). Anaemia of chronic disease was the most prevalent iron disorder (39%), regardless of ferritin cut-off. Using a higher ferritin cut-off of 30 µg/L significantly increased the detection rates of both IDA and iron deficiency without anaemia (*p* < 0.001).

**Conclusion:**

This study suggests that a higher ferritin threshold (30 µg/L) might improve diagnosis of iron disorders in settings with high inflammatory diseases. Further studies are needed to refine thresholds. Local guidelines should be adjusted to consider higher ferritin cut-offs, and longitudinal studies are recommended to evaluate long-term outcomes.

**What this study adds:**

This study confirms the use of higher ferritin cut-offs for enhanced detection of iron deficiency states. The findings also emphasise the ongoing need for establishing simple, standardised, and accurate methods for iron status classification.

## Introduction

Iron deficiency is the most common nutritional disorder worldwide, and more prevalent in developing countries.^1,2,3^ Pregnant women, young children, and women of reproductive age are at higher risk.^4,5^ Iron deficiency refers to decreased or depleted iron stores, which precedes the development of ineffective haematopoiesis resulting in iron deficiency anaemia (IDA).^4^ Iron deficiency can also manifest as part of complex pathological processes seen in chronic diseases, where it is called functional iron deficiency. Without treatment, iron deficiency can cause significant morbidity and mortality, making an accurate and timely diagnosis important.^6^

Accurate determination of iron status is often difficult and complex. The gold standard for diagnosis of iron deficiency is a bone marrow biopsy demonstrating the absence of stainable iron.^2,7^ However, since this approach is costly and invasive, biochemical tests are more frequently utilised for diagnosis. A working group from the World Health Organization has recommended five biomarkers for the work-up of iron deficiency, namely haemoglobin, mean cell volume (MCV), zinc protoporphyrin, soluble transferrin receptor, and serum ferritin.^8^

Although haemoglobin concentration, MCV and ferritin are readily available in South Africa, zinc protoporphyrin and soluble transferrin receptor are not commonly measured. Specifically, in our setting at Chris Hani Baragwanath Academic Hospital, the markers available to assess iron status are haemoglobin concentration, MCV, serum iron, transferrin with transferrin saturation (TSAT), and serum ferritin. The diagnosis of the various pathological processes rests on the integrated interpretation of these tests.^2,7,9,10^

Interpretation of these biochemical parameters with a subsequent diagnosis is not always simple, as the effect of inflammation is a confounding factor that needs to be considered. Chronic inflammation is associated with production of cytokines, which blunts the erythropoietin response. This results in a disruption of normal iron homeostasis and causes increased uptake of iron, with subsequent retention thereof in the reticuloendothelial system. The outcome is iron-restricted erythropoiesis, followed by the development of anaemia of chronic disease (ACD) with an elevated ferritin, low haemoglobin, and decreased TSAT.^2,3,11^ Furthermore, inflammation induces pro-inflammatory cytokines such as interleukin 6, which increases hepcidin production. Hepcidin acts as a regulator of iron homeostasis by mediating the internalisation of iron and degradation of ferroportin, the iron exporter. This results in decreased iron release from enterocytes and macrophages and, subsequently, to lower plasma iron levels than required to meet the body’s needs. In addition, serum ferritin is significantly elevated during inflammation.^12,13,14,15^

In the South African setting, a national health and demographic survey found that as much as a third of South African women of reproductive age are anaemic based on point-of-care haemoglobin measurement, although iron status was not assessed.^6^ A meta-analysis found that the prevalence of anaemia in women of reproductive age ranged from 22.0% to 44.0%, compared to iron deficiency which ranged from 7.7% to 19.0%, and IDA, specifically, which ranged from 9.7% to 10.5%, utilising a ferritin cut-off of < 15 µg/L.^16^ Furthermore, it was found that IDA in pregnant patients resulted in an increased risk for premature birth, thus emphasising the importance of a correct and timely diagnosis.^17^ In the general South African population, 12.6% of participants had anaemia, compared to the high prevalence of 39.8% for iron deficiency, in this case specifically using the higher ferritin cut-off of < 30 µg/L for improved sensitivity.^4^ The distribution of anaemia between the sexes indicated a much higher prevalence of 18.3% in women compared to 2.9% in men.^4^ Despite these assessments, the iron status of hospital-attending patients in the South African context has not been studied.

A 2016 study in Cape Town, South Africa, found ACD to be the predominant cause of anaemia in patients with HIV and tuberculosis, seen in over 95% of their study population, also utilising the higher ferritin cut-off of < 30 µg/L.^18^ Importantly, this study was more extensive in the usage of other biochemistry of iron status such as soluble transferrin receptor, as well as infective markers such as C-reactive protein, which likely improved the sensitivity. The high prevalence of chronic inflammatory conditions like HIV, as well as chronic kidney disease, together with the presence of inflammation in hospitalised patients, increases the prevalence of ACD.^19,20^ This raises the question on the iron status of an inpatient population, and on the diagnostic impact of coexisting ACD and iron deficiency.

Evidently, the cut-off used for ferritin for the diagnosis of iron deficiency is not harmonised, with most studies suggesting using < 30 µg/L, and others, < 15 µg/L.^2,10^ Clinicians at our site frequently rely on a ferritin cut-off value of < 15 µg/L for the diagnosis of IDA, based on the World Health Organization guidelines,^8^ and this is applied universally, regardless of sex. The question remains whether this international guideline is applicable to the South African population and, more specifically, to a hospital-attending cohort. To our knowledge, there are no local studies that have investigated the effect on the classification of iron status using these different cut-offs to date.

Given the aforementioned context, there is a need to investigate the diagnostic utility of these available biochemical parameters for iron status evaluation in the South African milieu. Thus, the aims of this retrospective study were to analyse and categorise the iron profiles in a South African adult population treated at a tertiary hospital setting, and to evaluate how various ferritin thresholds influence the identification of iron deficiency.

## Methods

### Ethical considerations

Ethical approval to conduct the study was obtained from the Human Research Ethics Committee of the University of the Witwatersrand (reference no.: M210512). Patients’ informed consent was not required due to the retrospective nature of the study. Patient data were de-identified to maintain patient privacy and anonymity. Data were securely stored on a password-protected Excel spreadsheet (Office 2016, Microsoft, Redmond, Washington, United States), accessible only to the investigators of the study. The research was conducted adhering to the ethical principles of the Declaration of Helsinki (2013).

### Study design and setting

This study employed a retrospective cross-sectional design analysing laboratory data from patients attending Chris Hani Baragwanath Academic Hospital in Soweto, South Africa. Pathology services are provided primarily by the National Health Laboratory Services, which serves 80% of the population in the public sector health system.

### Data collection

Data for the period from 01 October 2020 to 31 March 2021 were obtained from the National Health Laboratory Services Corporate Data Warehouse after mining from the Laboratory Information System (TrackCare Lab, InterSystems Corporation, Cambridge, Massachusetts, United States). The study included results from adult patients (> 18 years) attending Chris Hani Baragwanath Academic Hospital who had the following parameters: sex, haemoglobin, MCV, iron, transferrin, TSAT, and ferritin. Patient results with missing data for age, sex, haemoglobin, ferritin, or TSAT were excluded.

### Laboratory analysis

The iron profile analyses (serum iron, transferrin and ferritin) were conducted on the Roche **cobas**^®^ 8000 automated chemistry analyser (Roche Diagnostics, Mannheim, Germany). Iron was measured using the FerroZine colourimetric method without deproteinisation. Iron reacts with FerroZine to form a coloured complex which is measured photometrically. Transferrin and ferritin were measured using immunoturbidimetric methods utilising anti-human transferrin and anti-human ferritin antibodies, respectively. Transferrin saturation was calculated according to the formula: % saturation = (iron / [transferrin x 25.6]) × 100%.

The haematological parameters (haemoglobin, MCV) were measured using the Sysmex XN10/XN20 series automated haematology analyser (Sysmex Corporation, Kobe, Japan). Haemoglobin was measured spectrophotometrically using a non-cyanide sodium lauryl sulphate. Mean cell volume was calculated as follows: haematocrit % × 10 ÷ red blood cell count. The National Health Laboratory Services laboratory at Chris Hani Baragwanath Academic Hospital maintains accreditation status with the relevant governing bodies and participates in external quality assurance schemes for ongoing independent quality assessment.

### Data processing

Data included in the study were classified into IDA, ACD, iron deficiency with ACD (iron deficiency + ACD), iron deficiency without anaemia (IDWA), and iron replete using pre-defined criteria and two different cut-offs for ferritin (Table 1). Haemoglobin and ferritin levels were used as hard criteria for classification, while TSAT was used primarily as an adjunct. Results that did not fall into these categories using the criteria were classified as indeterminate.

**TABLE 1:** Criteria for iron status classification, Johannesburg, South Africa, 01 October 2020 to 31 March 2021.

Parameter	Sex	Iron deficiency anaemia	Anaemia of chronic disease	Iron deficiency + anaemia of chronic disease	Iron deficiency without anaemia	Iron replete
Haemoglobin (g/dL)†:	Male	< 13	< 13	< 13	≥ 13	≥ 13
	Female	< 12	< 12	< 12	≥ 12	≥ 12
Ferritin (μg/L):	Female	< 15 or <30	≥ 100‡	15–100 or 30–100	< 15 or < 30	15–150
	Male	< 15 or <30	≥ 100	15–100 or 30–100	< 15 or < 30	30–400
TSAT (%)	-	≤ 16§	≤ 16 or 17–45	≤ 16 or 17–45	≤ 16	17–45

TSAT, transferrin saturation.

† , The values for haemoglobin in the assessment of anaemia are from the World Health Organization.

‡ , The values for ferritin in the assessment of anaemia of chronic disease are from Weiss and Goodnough.^11^

§ , The values for transferrin saturation for the assessment of iron deficiency anaemia is from Beutler and Waalen.^9^

### Statistical analysis

Statistical analyses were performed using SPSS software 29.0.0.0 (IBM Corp. Armonk, New York, United States). All the data were non-parametric when the normality was evaluated using the Shapiro-Wilk test. Categorical data were presented using frequencies and percentages. Non-parametric data were presented as median (interquartile range). The Chi-squared test was used to compare categorical variables, while the Mann-Whitney *U* test was used to compare the medians of two groups of continuous data. For continuous data with three or more groups, the Kruskal-Wallis test with Bonferroni correction was employed to assess the differences in medians among the groups. A *p*-value of < 0.05 was considered statistically significant.

## Results

### Demographics

**FIGURE 1:**
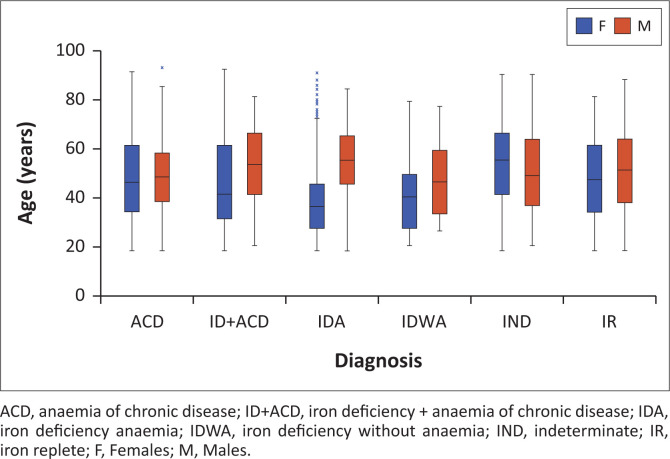
Age differences between male and female patients in different iron status groups defined using a ferritin 30 µg/L, Johannesburg, South Africa, 01 October 2020 to 31 March 2021.

Following the removal of duplicate episodes (*n* = 2249), results showing an age below 18 years (*n* = 248), results with missing demographic data (*n* = 48), and incomplete profiles (*n* = 3891), 3221 episodes from a total of 9657 episodes were included in the analysis (Table 2). There was a predominance of women with a ratio of 2.2:1. Women were also younger overall, with a median age of 42 years, compared to 50 years for men. This difference was statistically significant in the IDA (*p* < 0.001) and iron deficiency + ACD (*p* < 0.001) groups, but not for the ACD (*p* = 0.430), IDWA (*p* = 0.260), iron replete (*p* = 0.212) and indeterminate (*p* = 0.345) groups (Figure 1). The majority of requests originated from the inpatient population (62%), primarily from the medical (82%) and surgical departments (17%) for male patients, and from the medical (68%) and maternity (23%) departments for female patients (Online Supplementary Figure 1).

**TABLE 2:** Iron status of patients in Johannesburg, South Africa, 01 October 2020 to 31 March 2021.

Parameter	Total					Women					Men				
	*n*	%	Median	IQR	*p*	*n*	%	Median	IQR	*p*	*n*	%	Median	IQR	*p*
Profiles	3221	-	-		-	2201	68	-		-	1020	32	-		< 0.001*
Age (years)	-	-	45	33–60	-	-	-	42	31–59	-	-	-	50	38–61	< 0.001**
**Location**	-	-	-	-	-	-	-	-	-	< 0.001***	-	-	-	-	< 0.001***
Inpatient	1982	62	-	-	-	1231	56	-	-	-	751	74	-	-	-
Outpatient	1239	38	-	-	-	970	44	-	-	-	269	26	-	-	-
**Diagnosis (ferritin < 15 µg/L)**															
Iron deficiency anaemia	458	14	-	-	-	402	18	-	-	-	56	6	-	-	-
Anaemia of chronic disease	1285	39	-	-	-	712	32	-	-	-	573	56	-	-	-
Iron deficiency + anaemia of chronic disease	731	23	-	-	-	584	27	-	-	-	147	14	-	-	-
Iron deficiency without anaemia	14	1	-	-	-	14	1	-	-	-	0	0	-	-	-
Iron replete	315	10	-	-	-	178	8	-	-	-	137	13	-	-	-
Indeterminate	418	13	-	-	-	311	14	-	-	-	107	11	-	-	-
**Diagnosis (ferritin < 30 µg/L)**															
Iron deficiency anaemia	758	24	-	-	< 0.001****	645	29	-	-	< 0.001****	113	11	-	-	< 0.001****
Anaemia of chronic disease	1285	39	-	-	1.000****	712	32	-	-	1.000****	573	56	-	-	1.000****
Iron deficiency + anaemia of chronic disease	431	13	-	-	< 0.001****	341	16	-	-	< 0.001****	90	9	-	-	< 0.001****
Iron deficiency without anaemia	79	3	-	-	< 0.001****	70	3	-	-	< 0.001****	9	1	-	-	< 0.001****
Iron replete	304	10	-	-	0.672****	167	8	-	-	0.499****	137	13	-	-	1.000****
Indeterminate	364	11	-	-	0.062****	266	12	-	-	0.052****	98	10	-	-	0.461****

IQR, interquartile range.

* , *p*-value: Women vs men, Chi-squared test.

** , *p*-value: Women vs men, Mann-Whitney *U* test.

*** , *p*-value: Inpatients vs outpatients, Chi-squared test.

**** , *p*-values: Ferritin 30 µg/L vs ferritin 15 µg/L, Chi-squared test.

### Frequencies of iron disorders based on ferritin cut-off

Irrespective of the ferritin cut-off used for iron status classification, ACD was the most prevalent iron disorder (overall = 39%) in this study. In contrast, the frequency of patients with IDA, iron deficiency + ACD, IDWA and iron replete varied depending on the ferritin cut-off employed for the classification (Table 2). The use of the higher ferritin cut-off of 30 µg/L results in a statistically significant increase in the frequency of IDA (*p* < 0.001) and IDWA (*p* < 0.001). Utilising this higher ferritin cut-off in combination with a TSAT of <16% improved the differentiation of IDWA from the indeterminate group, reducing the frequency of the latter from 11% to 5% (Online Supplementary Figure 2). The lower ferritin cut-off also precludes the identification of IDWA in the male group. The iron deficient states, IDA, IDWA, and iron deficiency + ACD were more frequent in female patients. Less than 15% of the study group could not be classified into any iron status category as per the criteria in Table 1 and were therefore classified as indeterminate.

### Distributions of parameters across different iron status groups

The pattern of distribution of parameters in the IDA group remained consistent across the two different ferritin cut-offs employed (Table 3). Lower levels of iron, TSAT and MCV were found in the IDA and iron deficiency + ACD groups compared to other groups. The highest ferritin levels were present in the ACD (median = 554 µg/L [258–1156]) and indeterminate (median = 170 µg/L [58–361]) groups. The iron replete and indeterminate groups were statistically similar in the distribution of parameters, except for transferrin and ferritin (Table 3).

**TABLE 3:** Distribution of parameters according to diagnosis using two different ferritin cut-offs, Johannesburg, South Africa, 01 October 2020 to 31 March 2021.

Parameter	Diagnosis												*p* **
	Iron deficiency anaemia		Anaemia of chronic disease		Iron deficiency + anaemia of chronic disease		Iron deficiency without anaemia		Iron replete		Indeterminate		
	Median	IQR	Median	IQR	Median	IQR	Median	IQR	Median	IQR	Median	IQR	
**Ferritin < 15 µg/L**													
Ferritin (µg/L)	9*	6–12*	554*	258–1156*	36*	21–58*	12*	10–13*	95	59–142	170*	58–361*	< 0.001
TSAT (%)	4*	3–6*	21	13–32	9*	6–15*	12*	7–15*	24	19–30	15	12–23	< 0.001
Transferrin (g/L)	3.71*	3.26–4.19*	1.65*	1.29–2.01*	2.95*	2.40–3.57*	3,22	2.97–3.53	2.61	2.31–2.91	2.58*	2.15–3.03*	< 0.001
Iron (µmol/L)	3.7*	2.8–5.2*	8.2*	4.9–14*	6.4*	4.3–10.5*	8.7*	6.4–13*	12.1	16.0–19.9	8.0	10.4–13.5	< 0.001
Haemoglobin (g/dL)	7.2*	5.5–8.5*	8.6*	6.9–10.3*	9.6*	7.7–10.9*	12.6	12.1–12.9	14.0	13.2–15.1	13.6	12.8–14.4	< 0.001
MCV (fL)	72.8*	67.1–79.1*	91.6*	84.9–97.7*	85.3*	77.6–92.5*	86.4	79.5–92.6	94.1	89.5–98.2	92.8	87.3–98.2	<0.001
**Ferritin < 30 µg/L**													
Ferritin (µg/L)	12*	8–18*	554*	258–1156*	52*	39–74*	22*	18–27*	100	63–143	196*	86–414*	< 0.001
TSAT (%)	5*	4–7*	21	13–32	11*	7–18*	12*	10–17*	25	19–31	16	13–24	< 0.001
Transferrin (g/L)	3.59*	3.09–4.17*	1.65*	1.29–2.01*	2.64	2.19–3.23	3.12*	2.86–3.43*	2.59	2.30–2.91	2.52*	2.08–2.94*	< 0.001
Iron (µmol/L)	4.3*	3.1–6.6*	8.2*	4.9–14*	7.1*	4.6–11.4*	10.1*	8–13.7*	16.2	12.1–20.2	10.5	8.0–13.7	< 0.001
Haemoglobin (g/dL)	7.8*	6.2–9.6*	8.6*	6.9–10.3*	9.9*	8.1–11.10*	13.1	12.4–13.8	14.1	13.2–15.2	13.6	12.8–14.5	< 0.001
MCV (fL)	76.1*	69.7–84.1*	91.6*	84.9–97.7*	88.4*	79.6–94.8*	91.3	86.3–95.2	94.3	89.6–97.8	92.9	87.4–98.5	< 0.001

IQR, interquartile range; TSAT, transferrin saturation; MCV, mean cell volume.

* , *p* < 0.05 vs iron replete.

** , Kruskal-Wallis test.

## Discussion

Employing a multi-marker approach for the assessment of iron status, we classified 3221 iron profiles using haemoglobin, serum ferritin with two different cut-offs, and TSAT. Consistent with another study conducted in 2020 in patients attending a hospital in Italy,^21^ ACD (39%) was the most common iron disorder in our setting, irrespective of the ferritin threshold (15 µg/L vs 30 µg/L). Using different thresholds did not affect the frequency, as the diagnostic criterion employed was a serum ferritin concentration > 100 µg/L, and the observed median ferritin levels (554 µg/L, interquartile range: 258–1156) were substantially elevated. The predominance of ACD in our setting can be attributed to the high burden of pro-inflammatory communicable and non-communicable disorders such as HIV, chronic kidney disease, type 2 diabetes, and metabolic syndrome. Anaemia of chronic disease results from a cytokine-mediated disruption of iron homeostasis, affecting red blood cell survival, erythropoiesis, and iron efflux from macrophages. The elevations in serum ferritin seen in these patients stem from stimulation of the divalent metal transporter 1 with increased intracellular iron shifts.^11,22^ The risk of developing ACD increases with advancing age, which corresponds with our observation that ACD was the most frequent iron disorder in the elderly (38.%). Among all the categories in our study, patients in the ACD group had the lowest levels of transferrin (median = 1.65 µg/L, interquartile range: 1.29–2.01). This observation can be explained by the hepatic inhibition of transferrin production as a consequence of the ongoing inflammatory process.^23^

An examination of the impact of applying the two distinct ferritin cut-offs on the distribution of patients between iron status categories revealed a noticeable shift in all groups except for ACD. When using a higher cut-off of 30 µg/L, patients initially categorised as iron deficiency + ACD, iron replete, or indeterminate were re-classified as iron deficiency and IDWA, with a 1.7-fold increase for iron deficiency, and a 5.6-fold increase for IDWA. The cut-off of 30 µg/L has been shown to have significantly higher sensitivity (98%) when compared to a serum ferritin of 15 µg/L, which is highly specific but lacks sensitivity for recognising iron deficiency, as shown in studies conducted in 1998 in America and 1993 in Sweden.^24,25^ This may explain the higher prevalence of profiles identified as indicative of iron deficient states when using 30 µg/L as a serum ferritin threshold. The use of a higher threshold could inadvertently lead to an increase in false positives, but the primary advantage is the increased detection rate of cases that might otherwise go undetected when utilising the lower cut-off of 15 µg/L, which may include up to 50% of true cases of IDA.^26^ Within our clinical setting, there is widespread adoption of the World Health Organization-recommended ferritin cut-off value of 15 µg/L for the diagnosis of IDA. However, potential confusion may arise due to the presence of method-specific (and potentially sex-specific) reference intervals for ferritin reported by the National Health Laboratory Services Laboratory Information System. It is crucial to recognise that these reference intervals are not directly applicable to the diagnosis of all iron status disorders. Instead, they represent the range of ferritin values observed in a healthy population.

It is important to identify and treat iron deficiency, even in the absence of anaemia (normal haemoglobin levels), in order to minimise any potential adverse consequences, such as reduced cognitive function, poor performance, and poorer outcomes across a range of medical conditions that may occur in IDWA.^27,28,29^ Although previous research indicates that the frequency of IDWA may be twice as high as that of IDA,^30^ our study population produced a lower than expected frequency (< 5% using either ferritin cut-off), likely because our group was primarily made up of patients attending a hospital or hospitalised, where IDA and ACD are expectedly more common. Despite our low frequency of IDWA (3%) being in sharp contrast to that identified in a healthy outpatient population in Lebanon in 2020 (57.5% using ferritin < 30 µg/L),^31^ it remains consistent with the established sex differences, where female patients exhibit a higher tendency for iron deficiency.^31^ The differences in prevalence estimates may also be due to lack of consistency in definitions and parameters, making it challenging to accurately identify IDWA.^28^ The common practice is relying on a normal haemoglobin combined with a low ferritin in a patient with suggestive clinical symptoms, but there is a lack of guidance for diagnosing IDWA in patients with disorders known to affect ferritin levels. As recommended by Al-Naseem et al. from England in 2021,^28^ when using a low TSAT as a diagnostic criterion in patients where ferritin levels are elevated, our study showed the prevalence of IDWA increases to 8% (ferritin cut-off 15 µg/L) and 9% (ferritin cut-off 30 µg/L). These results imply that the inclusion of TSAT may reclassify patients who, according to traditional parameters, would otherwise be classified as indeterminate using our criteria. It is important to acknowledge that TSAT is a ratio of serum iron to transferrin or total iron binding capacity, and is therefore susceptible to variations in either of these parameters.^32^ Serum iron levels are strongly influenced by dietary consumption and show pronounced diurnal fluctuations, while conditions such as chronic illness and malnutrition suppress the synthesis of transferrin.^33^ This is supported by evidence from research conducted in 2022 on Kenyan patients that TSAT (< 20%) shows a relatively low predictive value (31.5%) in identifying IDWA.^34^

Mixed aetiology anaemia, iron deficiency + ACD, is characterised by the co-occurrence of true iron deficiency in patients with ACD. The underlying mechanism is thought to be chronic blood loss associated with a disorder with persistent inflammatory activity.^35^ It is important to accurately diagnose true iron deficiency in ACD patients in order to prevent delayed iron replacement and failure to identify the underlying cause of blood loss.^36,37^ The substantial overlap in conventional blood parameters makes it difficult to distinguish between IDA, iron deficiency + ACD, and ACD. A limit of < 100 µg/L has been suggested as a diagnostic criterion for iron deficiency + ACD to account for the inflammation-induced elevation in ferritin.^14^ In our study, using this cut-off, iron deficiency + ACD ranked second behind ACD (defined by ferritin < 15 µg/L) as the most common iron disorder. It is interesting to note however, that a 2021 study conducted in gastroenterology patients in the United Kingdom^35^ has shown that non-traditional markers such as hepcidin, soluble transferrin receptor (sTfR), sTfR/log ferritin index, and reticulocyte haemoglobin equivalent, can be used to correctly reclassify a substantial proportion of patients in this group as either IDA or ACD alone, indicating that using only traditional markers may overstate the true prevalence of iron deficiency + ACD.^37^ However, these markers are not without limitations. For instance, the soluble transferrin receptor exhibits an inverse relationship with tissue iron availability and thus is elevated in iron deficiency states. However, due to its correlation with erythropoiesis, its levels also increase in response to enhanced erythropoietic activity, which poses a limitation.^32^ While the sTfR/log ferritin index has been proposed to improve diagnostic accuracy in anaemia classification compared to sTfR alone, the routine use of both soluble sTfR and the sTfR/log ferritin index remains limited due to cost considerations and significant analytical challenges. These challenges include the lack of standardisation in assay methodologies as well as diagnostic thresholds.^37,38^

In our study, the observed parameters in patients with IDA form a classic picture of microcytic anaemia, hypoferraemia, low ferritin levels, decreased TSAT percentages, and elevated transferrin levels. Interestingly, regardless of the cut-off, median IDA ferritin levels remained less than 15 µg/L. Mean cell volume was not used in this study as a diagnostic criterion for classification, but its analysis showed that subjects with iron deficiency (IDA, iron deficiency + ACD, and IDWA) tended to have lower median values than subjects with ACD, iron replete, and indeterminate categories. Notably, the median MCV for only the IDA group (72.8 fL, interquartile range: 67.1–79.1) fell below the reference range in our study. Studies show that up to 40% of IDA patients may have a normal MCV, thus its usefulness as a stand-alone diagnostic tool for iron deficiency is very limited.^39^

Lastly, there was a subset of profiles in our study that could not be categorised into any of the pre-defined groups according to our specified criteria and were deemed indeterminate. This unclassifiable group was predominantly characterised by profiles with normal haemoglobin, elevated serum ferritin, and borderline low TSAT levels, and is suggestive of underlying inflammation potentially affecting these parameters. It is noteworthy that a sizeable number of the profiles would be re-classified as IDWA had the TSAT been used in cases of elevated ferritin levels, decreasing the frequency of this group from 13% to 5% (ferritin < 15 µg/L) and 11% to 5% (ferritin < 30 µg/L).

### Limitations

As a retrospective study, this analysis of laboratory data is inherently limited by the lack of information on clinical diagnoses and potentially incomplete data, which may impact the interpretability of the findings. The lack of a comparison to the gold standard diagnostic test, a bone marrow biopsy, made it impossible to verify the accuracy of the IDA classification using ferritin. The lack of inflammatory marker data in this study restricts our ability to incorporate them directly into the analysis of inflammatory conditions groups and the impact on ferritin levels. Specifically, this data cohort was obtained during the coronavirus 2019 pandemic, which could have affected results due to the effect of inflammation on ferritin and transferrin concentration. While a range of ferritin cut-off values have been proposed for the diagnosis of ACD, we opted to utilise a cut-off of 100 µg/L. This specific value aligns with the established threshold for initiating iron replacement therapy in patients with confirmed IDA.^11^ Our selection of a TSAT < 16% for iron status classification differs from the < 20% cut-off employed by some authors, potentially affecting direct comparability of prevalence to other studies.^33^

### Conclusion

In the absence of an acceptable non-invasive gold standard marker for iron deficiency, a ferritin cut-off of 30 µg/L might be a suitable threshold for iron deficiency assessment. While this approach is likely to result in a small increase in false-positive diagnoses, more true iron deficiency cases will be identified compared to a lower ferritin cut-off of 15 µg/L. Furthermore, considering the potential underdiagnosis of IDWA based on existing literature, we recommend screening for iron deficiency using ferritin in patients with normal haemoglobin who present with suggestive clinical symptoms. Our findings highlight the difficulties in accurately assessing iron status using traditional laboratory tests. Non-standardised ferritin cut-offs, suboptimal test performance in inflammatory settings, and considerable overlap in marker profiles between iron deficiency and other conditions contribute to these challenges. Further investigation into the diagnostic accuracy, as well as the feasibility of employing novel markers in our setting, is warranted.

## References

[1] Thomas C, Thomas L. Biochemical markers and hematologic indices in the diagnosis of functional iron deficiency. Clin Chem. 2002;48(7):1066–1076. 10.1093/clinchem/48.7.1066 12089176

[2] Brugnara C. Iron deficiency and erythropoiesis: New diagnostic approaches. Clin Chem. 2003;49(10):1573–1578. 10.1373/49.10.1573 14500582

[3] Camaschella C. Iron-deficiency anemia. N Engl J Med. 2015;372(19):1832–1843. 10.1056/NEJMra1401038 25946282

[4] Phatlhane DV, Zemlin AE, Matsha TE, et al. The iron status of a healthy South African adult population. Clin Chim Acta. 2016;460:240–245. 10.1016/j.cca.2016.06.019 27339094

[5] Peyrin-Biroulet L, Williet N, Cacoub P. Guidelines on the diagnosis and treatment of iron deficiency across indications: A systematic review. Am J Clin Nutr. 2015;102(6):1585–1594. 10.3945/ajcn.114.103366 26561626

[6] Stoltzfus RJ. Iron deficiency: Global prevalence and consequences. Food Nutr Bull. 2003;24(4 Suppl.):S99–S103. 10.1177/15648265030244S206 17016951

[7] Dignass A, Farrag K, Stein J. Limitations of serum ferritin in diagnosing iron deficiency in inflammatory conditions. Int J Chronic Dis. 2018;2018:1–11. 10.1155/2018/9394060 PMC5878890 29744352

[8] World Health Organization. WHO guideline on use of ferritin concentrations to assess iron status in individuals and populations [homepage on the Internet]. Geneva: World Health Organization; 2020 [cited 2023 Aug 02]. Available from: https://www.who.int/publications/i/item/9789240000124 33909381

[9] Beutler E, Waalen J. The definition of anemia: What is the lower limit of normal of the blood hemoglobin concentration? Blood. 2006;107(5):1747–1750. 10.1182/blood-2005-07-3046 16189263 PMC1895695

[10] World Health Organization. Serum ferritin concentrations for the assessment of iron status in individuals and populations: Technical brief [homepage on the Internet]. Geneva: World Health Organization; 2020 [cited 2023 Aug 23]. Available from: https://www.who.int/publications/i/item/9789240008526

[11] Weiss G, Goodnough LT. Anemia of chronic disease. N Engl J Med. 2005;352:1011–1023. 10.1056/NEJMra041809 15758012

[12] Cappellini MD, Comin-Colet J, De Francisco A, et al. Iron deficiency across chronic inflammatory conditions: International expert opinion on definition, diagnosis, and management. Am J Hematol. 2017;92(10):1068–1078. 10.1002/ajh.24820 28612425 PMC5599965

[13] Ganz T. Systemic iron homeostasis. Physiol Rev. 2013;93:1721–1741. 10.1152/physrev.00008.2013 24137020

[14] Weiss G, Ganz T, Goodnough LT. Anemia of inflammation. Blood. 2019;133(1):40–50. 10.1182/blood-2018-06-856500 30401705 PMC6536698

[15] Archer NM, Brugnara C. Diagnosis of iron-deficient states. Crit Rev Clin Lab Sci. 2015;52(5):256–272. 10.3109/10408363.2015.1038744 26292073

[16] Turawa E, Awotiwon O, Dhansay MA, et al. Prevalence of anaemia, iron deficiency, and iron deficiency anaemia in women of reproductive age and children under 5 years of age in South Africa (1997–2021): A systematic review. Int J Environ Res Public Health. 2021;18(23):12799. 10.3390/ijerph182312799 34886524 PMC8656986

[17] Symington EA, Baumgartner J, Malan L, et al. Maternal iron-deficiency is associated with premature birth and higher birth weight despite routine antenatal iron supplementation in an urban South African setting: The NuPED prospective study. PLoS One. 2019;14(9):e0221299. 10.1371/journal.pone.0221299 31479449 PMC6719862

[18] Kerkhoff AD, Meintjes G, Opie J, et al. Anaemia in patients with HIV-associated TB: Relative contributions of anaemia of chronic disease and iron deficiency. Int J Tuberc Lung Dis. 2016;20(2):193–201. 10.5588/ijtld.15.0558 26792471 PMC6371921

[19] De Santis GC, Brunetta DM, Vilar FC, et al. Hematological abnormalities in HIV-infected patients. Int J Infect Dis. 2011;15(12):e808–e811. 10.1016/j.ijid.2011.08.001 21880530

[20] Braga F, Infusino I, Dolci A, Panteghini M. Soluble transferrin receptor in complicated anemia. Clin Chim Acta. 2014;431:143–147. 10.1016/j.cca.2014.02.005 24525213

[21] Randi ML, Bertozzi I, Santarossa C, et al. Prevalence and causes of anemia in hospitalized patients: Impact on diseases outcome. J Clin Med. 2020;9(4):950. 10.3390/jcm9040950 32235484 PMC7230611

[22] Madu AJ, Ughasoro MD. Anaemia of chronic disease: An in-depth review. Med Princ Pract. 2017;26(1):1–9. 10.1159/000452104 27756061 PMC5588399

[23] Peng YY, Uprichard J. Ferritin and iron studies in anaemia and chronic disease. Ann Clin Biochem. 2017;54(1):43–48. 10.1177/0004563216675185 27701066

[24] Mast AE, Blinder MA, Gronowski AM, Chumley C, Scott MG. Clinical utility of the soluble transferrin receptor and comparison with serum ferritin in several populations. Clin Chem. 1998;44(1):45–51. 10.1093/clinchem/44.1.45 9550557

[25] Hallberg L, Bengtsson C, Lapidus L, Lindstedt G, Lundberg P-A, Hultén L. Screening for iron deficiency: An analysis based on bone-marrow examinations and serum ferritin determinations in a population sample of women. Br J Haematol. 1993;85(4):787–798. 10.1111/j.1365-2141.1993.tb03225.x 7918045

[26] Daru J, Colman K, Stanworth SJ, et al. Serum ferritin as an indicator of iron status: What do we need to know? Am J Clin Nutr. 2017;106:1634–1643. 10.3945/ajcn.117.155960 PMC5701723 29070560

[27] Rattehalli D, Pickard L, Tselepis C, Sharma N, Iqbal TH. Iron deficiency without anaemia: Do not wait for the haemoglobin to drop? Health Policy Technol. 2013;2(1):45–58. 10.1016/j.hlpt.2012.12.005

[28] Al-Naseem A, Sallam A, Choudhury S, Thachil J. Iron deficiency without anaemia: A diagnosis that matters. Clin Med (Lond). 2021;21(2):107–113. 10.7861/clinmed.2020-0582 33762368 PMC8002799

[29] Soppi ET. Iron deficiency without anemia – A clinical challenge. Clin Case Rep. 2018;6(6):1082–1086. 10.1002/ccr3.1529 29881569 PMC5986027

[30] Simic S, Karczewski M, Klapdor S, et al. Effectiveness of low-dose iron treatment in non-anaemic iron-deficient women: A prospective open-label single-arm trial. Swiss Med Wkly. 2023;153(5):40079. 10.57187/smw.2023.40079 37229775

[31] Abuaisha M, Itani H, El Masri R, Antoun J. Prevalence of iron deficiency (ID) without anemia in the general population presenting to primary care clinics: A cross-sectional study. Postgrad Med. 2020;132(3):282–287. 10.1080/00325481.2020.1715701 31933400

[32] Rohr M, Brandenburg V, Brunner-La Rocca HP. How to diagnose iron deficiency in chronic disease: A review of current methods and potential marker for the outcome. Eur J Med Res. 2023;28(1):15. 10.1186/s40001-022-00922-6 36617559 PMC9827648

[33] Fletcher A, Forbes A, Svenson N, Wayne Thomas D. Guideline for the laboratory diagnosis of iron deficiency in adults (excluding pregnancy) and children. Br J Haematol. 2022;196(3):523–529. 10.1111/bjh.17900 34693519

[34] Omuse G, Chege A, Kawalya DE, Kagotho E, Maina D. Ferritin and its association with anaemia in a healthy adult population in Kenya. PLoS One. 2022;17(10):e0275098. 10.1371/journal.pone.0275098 36240192 PMC9565368

[35] Svenson N, Bailey J, Durairaj S, Dempsey-Hibbert N. A simplified diagnostic pathway for the differential diagnosis of iron deficiency anaemia and anaemia of chronic disease. Int J Lab Hematol. 2021;43(6):1644–1652. 10.1111/ijlh.13666 34288431

[36] Goodnough LT, Nemeth E, Ganz T. Detection, evaluation, and management of iron-restricted erythropoiesis. Blood. 2010;116(23):4754–4761. 10.1182/blood-2010-05-286260 20826717

[37] Skikne BS, Punnonen K, Caldron PH, et al. Improved differential diagnosis of anemia of chronic disease and iron deficiency anemia: A prospective multicenter evaluation of soluble transferrin receptor and the sTfR/log ferritin index. Am J Hematol. 2011;86(11):923–927. 10.1002/ajh.22108 21812017

[38] Lyle AN, Budd JR, Kennerley VM, et al. Assessment of WHO 07/202 reference material and human serum pools for commutability and for the potential to reduce variability among soluble transferrin receptor assays. Clin Chem Lab Med. 2023;61(10):1719–1729. 10.1515/cclm-2022-1198 37071928 PMC13361177

[39] Bermejo F, García-López S. A guide to diagnosis of iron deficiency and iron deficiency anemia in digestive diseases. World J Gastroenterol. 2009;15(37):4638–4643. 10.3748/wjg.15.4638 19787826 PMC2754511

